# Gadolinium in Medical Imaging—Usefulness, Toxic Reactions and Possible Countermeasures—A Review

**DOI:** 10.3390/biom12060742

**Published:** 2022-05-24

**Authors:** Lennart Blomqvist, Gunnar F. Nordberg, Valeria M. Nurchi, Jan O. Aaseth

**Affiliations:** 1 Department of Molecular Medicine and Surgery, Karolinska Institutet, SE-17176 Stockholm, Sweden; lennart.k.blomqvist@ki.se; 2 Department of Medical Radiation Physics and Nuclear Medicine, Karolinska University Hospital, SE-17176 Stockholm, Sweden; 3 Division of Sustainable Health, Department of Public Health and Clinical Medicine, Umeå University, SE-90187 Umeå, Sweden; 4 Department of Life and Environmental Sciences, University of Cagliari, 09042 Cagliari, Italy; nurchi@unica.it; 5 Department of Research, Innlandet Hospital Trust, P.O. Box 104, N-2381 Brumunddal, Norway; jaol-aas@online.no; 6 Faculty of Health and Social Sciences, Inland Norway University of Applied Sciences, N-2418 Elverum, Norway

**Keywords:** gadolinium, chelates, gadolinium toxicity, gadolinium kinetics, side effects of gadolinium chelates, nephrogenic systemic fibrosis, gadolinium induced respiratory distress syndrome, contrast induced nephropathy, treatment of gadolinium toxicity

## Abstract

Gadolinium (Gd) is one of the rare-earth elements. The properties of its trivalent cation (Gd^3+^) make it suitable to serve as the central ion in chelates administered intravenously to patients as a contrast agent in magnetic resonance imaging. Such Gd-chelates have been used for more than thirty years. During the past decades, knowledge has increased about potential harmful effects of Gd-chelates in patients with severe renal dysfunction. In such patients, there is a risk for a potentially disabling and lethal disease, nephrogenic systemic fibrosis. Restricting the use of Gd-chelates in persons with severely impaired renal function has decreased the occurrence of this toxic effect in the last decade. There has also been an increasing awareness of Gd-retention in the body, even in patients without renal dysfunction. The cumulative number of doses given, and the chemical structure of the chelate given, are factors of importance for retention in tissues. This review describes the chemical properties of Gd and its medically used chelates, as well as its toxicity and potential side effects related to injection of Gd-chelates.

## 1. Introduction

Gadolinium (Gd, atomic mass 157.25, atomic number 64) is a soft silvery white metal that reacts with oxygen and water. In its ionic trivalent form gadolinium has seven unpaired electrons leading to a large inherent magnetic moment. At temperatures above 20 °C Gd is paramagnetic. Its electronic configuration confers a long spin relaxation time to its compounds. Gadolinium was identified in 1880 by spectroscopy of the mineral gadolinite by Jean de Marignac. He named the element as Gadolinium after the mineral gadolinite. This mineral is named after the Swedish/Finnish chemist Johan Gadolin, who discovered and characterized it in the 18th century.

**Figure 1:**
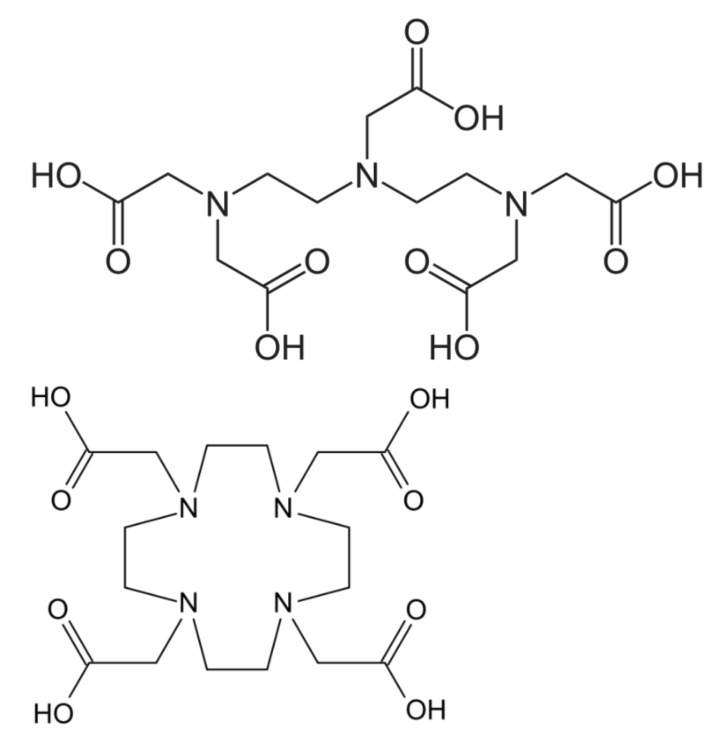
Chemical structure of a linear chelator, DTPA (**top**) and a macrocyclic chelator, DOTA (**bottom**), illustrating the ligands that bind to Gd. Macro-cyclic Gd(III) compounds are considered less likely to release free Gd(III) ions in the human body than linear chelates [1,2,3,4,5].

The Lanthanides (or lanthanoides) is a group of metals starting from the element lanthanum with atomic number 57 and including the 14 subsequent elements in the periodic table with numbers 58–71, thus, including gadolinium (Gd) with atomic number 64. In the great majority of its compounds, gadolinium, in ionic form, adopts the oxidation state +3. The electronic structure of the neutral gadolinium atom can be represented as [Xe]4f^7^5d^1^6s^2^. In its ionic state, Gd^+3^ uses the 6s^2^ and 5d¹ electrons for bonding. The seven unpaired electrons in 4f spherically symmetric orbital account for the strong paramagnetic effect. These electrons contribute to shorten the relaxation times of water molecules through exchange with the inner sphere coordinated water molecules in a very effective way. Gd^3+^ chelators used in contrast agents substitute the coordinated water molecules leaving only one of them bound, and this remaining molecule is characterized by an exchange rate more than two orders of magnitude less than that in the free Gd^3+^ ion. This has consequences on the amount of contrast agents that have to be used. Like most lanthanide ions, gadolinium(III) forms complexes with high coordination numbers. This tendency is illustrated by the practical use of polydentate chelating agents such as derivatives of DTPA (diethylenetriamine pentaacetic acid) and DOTA (tetraazacyclododecane tetraacetic acid), to bind gadolinium in chelates with relatively low toxicity [1] (Figure 1). Chelated derivatives of Gd-(DOTA), and also of Gd-(DTPA), are widely used in medicine as contrast agents in magnetic resonance imaging (MRI).

According to a recent survey of its global use, a total of about 10 million doses of Gd-chelates are administered annually [4]. This widespread use is due to the physical properties and presence of unpaired electrons, that make Gd(III), in the form of Gd chelates, particularly useful as gadolinium-based contrast agents (GBCAs) in magnetic resonance imaging (MRI).

The aim of the present review is to discuss routine medical use, and possible toxicity, of Gd-based contrast agents, and to highlight observed adverse reactions, in addition to reviewing research to avoid, or treat, toxic reactions.

## 2. Gadolinium in Medical Imaging

Gd in the form of Gd chelates is useful as a contrast agent in magnetic resonance imaging (MRI), due to its physical properties and presence of unpaired electrons in an inner shell of the Gd atom. The two main categories of GBCAs are the linear and the macrocyclic compounds (Figure 1). Macrocyclic GBCAs form cage like structures with Gd(III) enclosed in the cavity of the complex, e.g., in the DOTA complex. Such macrocyclic GBCAs have higher stability and are more inert in vivo than linear GBCAs [5]. The first human clinical injection of Gd-chelates was performed in 1988, and the use of Gd in clinical MRI examinations has increased over the years and now makes up almost half of all procedures [6]. The use of Gd-chelates shortens T1- and T2- relaxation times, where the shortening of T1- in T1-weighted imaging dominates.

Injection of a Gd-chelate intravenously in small doses (0.025–0.3 mmol/kg body weight), usually through a cubital vein, is especially used to study tissue perfusion and composition of interstitial spaces. During the first passage through the vascular system, a proportion of the agent passes across the vascular endothelium into the extracellular space [7]. After a few minutes, when the concentration of the agent within the vascular and extracellular space is practically equal, the so-called “equilibrium phase” is reached. These agents are usually referred to as extra-cellular agents. In the brain, the chelate is distributed into areas where there is a damaged blood-brain barrier. Most Gd-chelates are water-soluble and cleared from the body through glomerular filtration through the kidneys. A few dedicated agents are more or less fat-soluble and partly (3–50%) taken up in the liver by hepatocytes, and through the same transporter system as bilirubin carried to the biliary system and into the gut. These agents are usually referred to as “hepatocyte-specific” or “intra-cellular agents”. Clinically, except for the intracellular agents, the most commonly used GBCAs today are the macrocyclic water-soluble agents, due to their in vivo inertness.

In a wide range of clinical settings additional information is obtained by using contrast-enhanced imaging compared to non-enhanced imaging This is true both for dedicated organs, such as the brain, heart, breast, upper abdominal organs (liver, spleen, kidney, adrenals, pancreas), pelvic organs (uterus, prostate, urinary bladder, male and female genitalia), as well as for whole body applications, such as the vascular tree. Whenever it is possible and considered of importance to add clinically relevant information, imaging of a certain organ takes place by repeated scanning during the examination, dynamically, during and after injection, to obtain time resolved information of the biodistribution of the agent. This information can be used to add functional information about tissues, which is not obtained when images are obtained in the equilibrium phase.

When ordinating contrast agents in MRI it is important to make sure that the injection is likely to add clinically relevant information to non-enhanced imaging with an individual risk-benefit consideration based on current knowledge. In preparation for MRI, GBCAs are injected intravenously. The standard clinical dose for the majority of applications when an extracellular Gd-based contrast agent is injected intravenously, is 0.1 mmol/kg body weight [8]. In specific applications, such as MR-angiography or brain MRI for detection of metastases, doses up to 0.3 mmol/kg have been used. For these dedicated uses, Gd-chelates with higher molar concentration (1 mol Gd/L) are used, such as Gadobutrol (Gadovist, Bayer), which has twice the molar concentration of Gd compared to conventional extracellular agents [9]. In addition, there are two Gd-chelates that are specifically used in liver imaging, viz. Gadoxetic acid and Gd-BOPTA, that, beside renal excretion, are also taken up in the liver by hepatocytes and excreted through the biliary system. Gadoxetic acid has a molar concentration of 0.25 mol/L and is injected in a dose of 0.025 mmol/kg body weight [10]. Gd-BOPTA (Gadobenate dimeglumine) is characterized by a higher relaxivity than the other extracellular agents, has a molar concentration of of 0.5 mol/L and is injected in doses of 0.1 mmol/kg body weight. Gd-BOPTA is mainly used for liver imaging, but, due to its high relaxivity, is also useful for MRI-angiography [11]. GBCAs can also be injected directly into certain body cavities. The typical application is contrast-enhanced MRI-arthrography, when the contrast agent is injected directly into a joint (shoulder, hip, knee, ankle, elbow, wrist) to improve visualization of ligaments and cartilage. When injected into a cavity, a 0.5 mmol/m Gd-chelate has to be diluted in the order of 1 to 200 with 0.9% saline to obtain adequate shortening of T1 and T2 relaxation times [12]. Use of GBCAs in cavities and for arthrography is off-label use in many countries.

## 3. Pharmacokinetics and Toxicokinetics of Gd and Its Chelates

Wedeking and Tweedle [13] injected gadolinium acetate and gadolinium chelates in mice and found high levels in liver, kidneys and a much slower elimination of Gd-acetate than for Gd-chelates. Barnhart et al. [14] studied GdCl_3_ injected in rats and found high levels in liver, kidney and spleen and a slow elimination. The tissue retention is explained by the precipitation of Gd^3+^as gadolinium phosphate in blood and tissues as Gd^3+^ is insoluble at the pH of blood and tissues [15]. The side effects of gadolinium chelates when used in MRI, are, therefore, ascribed to release of free inorganic Gd-cations, which are particularly retained in the body in cases with severe kidney failure [16]. However, accumulation of gadolinium in the brain has been demonstrated by use of the ICP-MS-method in post mortem studies of patients without renal disease, in particular in the basal ganglia [17]. It was suggested, based on studies of the formation of Gd-ferritin nanoparticles in vitro, that the brain’s accumulation of Gd possibly represents ferritin nanoparticles [18]. It should be noted, however, that in other tissues accumulated gadolinium has been identified as gadolinium phosphate and Xia et al. [19] found deposits containing gadolinium phosphorus and calcium in brain tumor biopsies of patients given GBCAs. In vivo, it has been shown that the accumulation of gadolinium in basal ganglia and the dentate nucleus seems related both to the cumulative number of doses given as well as the type of Gd-chelate used [20].

Frenzel et al. [21] found that linear Gd chelates released more Gd^3+^ in human blood plasma in vitro than predicted from their stability constants, while the macrocyclic GBCAs were stable for 15 days. These findings are supported by other in vitro studies demonstrating a high stability of macrocyclic systems and a high sensitivity to transmetallation by Zn ions for other Gd-chelates [15,22,23]. Decomposition of injected GBCAs may cause release of inorganic ionic Gd(III), which may precipitate in the blood stream and other tissues. Gadolinium calcium phosphate deposits were identified in tissues. The above observations show that, besides the thermodynamic stability of the complexes, their kinetic inertness influences the toxicity of gadolinium chelates. Sherry et al. [24] clearly illustrate this point based on chemical considerations, observing what happens to a gadolinium complex dissolved in a solution at physiological pH containing phosphate anions. The high stability gadolinium chelates are in equilibrium with free gadolinium cations that, though at extremely low concentration, produce insoluble gadolinium phosphate. Chelate dissociation reconstitutes the necessary free gadolinium at thermodynamic equilibrium, at a faster rate depending on the kinetic features of the chelate, and this free gadolinium will produce further insoluble phosphate, and so on. Thus, thermodynamic stability of the contrast agent and its kinetic inertness are the fundamental parameters that counteract gadolinium toxicity. Solubility of inorganic Gd compounds is dependent on pH. While the free aquo-ion (for example from GdCl_3_) is freely soluble at pH 5 or lower, at physiological pH 7.4 it forms insoluble Gd^3+^ hydroxide colloids [25].

The intact water soluble chelates of Gd are efficiently excreted in the urine of patients with normal renal function, but pronounced tissue retention occurs in patients with impaired renal function. Therefore, restrictions have been introduced for the use of GBCAs in persons with impaired renal function. In recent years, increasing evidence indicates that there is some retention of a variable proportion of GdCAs, which can decompose into inorganic and toxic Gd(III) in various tissues, including in the brain, even in patients with normal renal function. Such retention is more pronounced for linear GdCAs than for macrocyclic ones. Frenzel et al. [9] performed studies in rats with normal kidney function and demonstrated increased retention of Gd after repeated injection of linear GdCAs, compared to macrocyclic compounds.

## 4. Toxic Effects of Gd-Compounds

The mechanism of toxicity of Gd-chelates is, to a large extent, unknown. However, it is known that unchelated Gd is highly toxic. In animals injected with gadolinium chloride, i.e., unchelated ionic gadolinium, there was hepatocellular and splenic toxicity with gadolinium calcium phosphate deposits in tissues [25]. When using GBCAs it is important that the chelated Gd is cleared by renal excretion before the chelate is dissociated. The half-life in humans of an intravenously injected clinical dose of an extracellular GBCA is about 90 min in healthy subjects [26], but can be significantly prolonged in subjects with impaired renal function, thereby increasing the risk that unchelated Gd is retained in the body. As suggested by Robert et al. [27], the term “gadolinium retention” or “gadolinium accumulation” is used when no precise mechanism is known for the retained gadolinium and “gadolinium deposition” when there is evidence that the deposits are made up of gadolinium phosphate that is very poorly eliminated [27]. There are a number of rather uncommon conditions related to Gd-chelate toxicity, such as contrast induced nephropathy, gadolinium deposition/retention disease (or gadolinium related symptoms) and nephrogenic systemic fibrosis.

For symptoms following GBCA administration unrelated to early onset (acute hypersensitivity and physiologic reactions) and late onset (nephrogenic systemic fibrosis), the American College of radiology has introduced the term “Symptoms Associated with Gadolinium Exposure” (SAGE) [28].

As for human observations, increasing knowledge about possible side effects from use of GBCAs have been obtained during recent decades. Some of these side effects, such as allergic and allergoid reactions including anaphylactic reactions, are general and may occur from use of almost any medical preparation. Since GBCAs are excreted by the kidneys, and removal of the agents is dependent on well-functioning kidneys, side effects such as nephrogenic systemic fibrosis caused by GBCA can be avoided if appropriate precautions are taken and/or if GBCAs are avoided in individuals at risk.

### 4.1. Acute Reactions to GBCAs

Acute reactions to GBCAs when injected during medical examinations are not common, but such reactions may occur if there is hypersensitivity, for example, and may present as anaphylactic reactions. For this reason, preparation for hypersensitivity reactions is part of the routine in departments where GBCAs are administered. It has been identified that some of the acute hypersensitivity reactions to these agents are likely to be IgE-mediated [29]. Acute respiratory effects have been reported in a few cases [30].

### 4.2. Contrast Induced Nephropathy

Iodinated contrast agents are known to present a risk of nephrotoxicity in certain instances. In an early phase of development of the GBCAs, there were suggestions that GBCAs could be considered as possible replacements for iodinated compounds as radiological contrast agents, but not until the nephrotoxicity of the GBCAs had been adequately examined. According to a literature review and evaluation by the Contrast Media Safety Committee of the European Society of Urogenital Radiology [31], data on animals show that GBCAs have more nephrotoxic potential than iodinated contrast media in equivalent X-ray attenuating doses; therefore, GBCAs should not replace iodinated contrast media in patients with renal insufficiency. In MRI procedures, GBCAs are given in doses that are 10–20 times lower than those used for iodinated contrast agents and such doses give rise to no, or minimal, nephrotoxicity. Nephrotoxicity does not usually occur due to GBCAs in regular use and there are only a few reports of contrast-induced nephropathy related to injection of GBCAs [32]. Due to the fact that this adverse event may occur in cases where GBCAs and iodinated contrast media have been given within a short time interval, guidelines for administration are designed to identify clinical risk factors, as well as warning against administration of GBCAs and iodinated contrast agents within 48 h of each other, unless clinically demanded.

### 4.3. Nephrogenic Systemic Fibrosis (NSF)

A condition named nephrogenic systemic fibrosis (NSF) may develop in patients with severe renal impairment that are exposed to GBCAs during MR examinations. This condition is characterized by gadolinium deposition in tissues (see also Section 5) and fibrotic infiltration of the skin and other organs [32]. NSF is potentially disabling and severe cases may be lethal. Concerns have been expressed for patients suffering from joint complaints. The gadolinium containing deposits in skin have been identified as gadolinium phosphate in autopsy samples from a case of NSF [33]. There was no evidence that the deposits contained GBCA [33]. However, further studies are desirable on the species of gadolinium deposited in skin and other tissues [34]. A consensus statement from the American College of Radiology (ACR) and the National Kidney Foundation in the US [35] used the ACR classification of GBCAs in relation to risk of NSF: Linear GBCAs are considered related to a high risk of NSF (Group I), with the exception of one compound (gadobenate dimeglumide). This compound is classified as low risk (Group II) together with the macrocyclic compounds. A third group (III) is stated to consist of the linear compound gadoxetate disodium, about which there is limited data regarding NSF risk. In the consensus document it was pointed out that the risk of NSF from Group II GBCAs in patients with acute kidney injury or eGFR less than 30 mL/1.73 m^2^ should be balanced against, and may outweigh, the risk of NSF [36]. The European Medical Agency, EMA [37], decided in 2017 to restrict the use of some linear GBCAs and to suspend the authorizations of others (see also Section 4.4). The recognition of NSF led to changed practices for use of the GBCAs and today the occurrence of NSF has been virtually eliminated.

### 4.4. Gadolinium Retention/Deposition

Recently, it was recognized that Gd is accumulated in tissues, such as skin, bone, kidneys and the brain, after repeated use of the linear GBCAs, even in patients with normal kidney function [15]. This was also concluded at a workshop [38], further concluding that an association between retention and symptoms reported by a small subset of patients given GBCAs has not been proven by scientific investigation [38]. Animal experiments have demonstrated brain accumulation of Gd after repeated dosing of the linear agents and, to some degree, also after repeated dosing of the macrocyclic compounds [39]. GBCAs used in MRI have caused a number of cases of NSF due to the Gd deposition, but restrictions in the use of these agents have decreased the number of such cases. In the last few years, brain accumulation of Gd has been identified as a potential problem [40], although clinical disease related to such accumulation has not been documented. As mentioned, the European Medical Agency, EMA [37], decided to restrict the use of some linear GBCAs and suspended the authorizations of others. These rules are now applicable in all EU member states. The macrocyclic compounds can continue to be used in their current indications but in the lowest doses that enhance images sufficiently, and only when non-enhanced body scans are not suitable.

### 4.5. Fetal Toxicity and the Use of GBCAs in Pregnancy

Khairinisa et al. [41] examined the offspring of pregnant mice i.v. injected on pregnancy day 15–19 with 2 mg/kg body weight of gadoterate meglumine (macrocyclic compound) or gadodiamide (linear compound); controls were injected with vehicle. Behavioral testing of offspring on day 70 showed anxiety-like behavior, disrupted motor coordination, impaired memory function, stimulated tactile sensitivity and decreased muscle strength in GBCA treated mice compared to controls, particularly in the gadodiamide-treated group. The study also showed increased Gd levels in the brains of the Gd-treated pups compared to controls. Mervak et al. [42] summarized available evidence of adverse effects on fetuses after Gd-assisted MRI in pregnant women and discussed how to use GBCAs in pregnant women. GBCAs pass the placental barrier to a certain extent, but after injection of GBCAs to pregnant non-human primates, the highest concentrations of Gd are in the placenta and the amniotic fluid with lower concentrations in fetal tissues [43]. In a retrospective study with 397 infants exposed to GBCAs in utero [44], the prevalence of stillbirth or neonatal deaths were slightly increased. Fetuses exposed to GBCAs during the first trimester demonstrated a slightly increased risk of childhood diagnosis of rheumatological, inflammatory or infiltrative skin conditions, compared to fetuses not undergoing MRI. There was no significant increase in NSF, and no increased risk of congenital anomalies (although the power of the study to show such effects was low). A more recent review [45] came to similar conclusions.

The United States FDA [46] classified gadolinium as a class C agent, meaning that animal studies have shown an adverse effect on the fetus and there are no adequate studies in humans. The FDA issued a class warning for GBCAs and noted that gadolinium may be retained in the body after administration, the linear GBCAs causing more retention than macrocyclic agents. GBCAs may be administered in a pregnant patient when benefits outweigh the potential risks. Given the potential for retention of gadolinium, retention characteristics should be considered and minimization of repetitive contrast-enhanced examinations in pregnant patients is recommended. In the European Union, the European Medicines Agency gives similar information. When there is a strong indication for contrast-enhanced MRI, the European Society of Urogenital Radiology recommends that the smallest possible dose of one of the most stable gadolinium chelates should be used.

## 5. Biological Monitoring

In clinical applications, patients are not routinely monitored after injection of GBCAs and in uncomplicated cases they are usually discharged after the MR-examination. If undesired reactions occur, either allergoid or allergic, the patients are usually kept within the hospital for monitoring until the reaction is under control or has vanished. If there is an unusual event with side effects related to impaired renal function after the combined use of GBCA and iodinated contrast media, this is usually discovered the same day as, or a few days after, the MR examination, and if so, the patient is usually kept within the hospital for hydration and monitoring of renal function.

Monitoring of tissue concentrations of gadolinium represents a possibility, for example by hair and serum determinations of gadolinium concentrations [47,48]. Such monitoring may not be justified at present in clinical work, because the scientific database is too limited. If further research provides sufficient data showing that these values form a reliable basis for estimating the risks of adverse effects in patients who are remitted to repetitive GBCA-assisted MRI examinations, hair and/or serum determinations may be of value in selected cases. However, at present, such monitoring is not adopted in clinical routines. There are a variety of methods to analyze Gd-concentrations in tissue samples, such as spatially resolved mass spectral based methods, combined with extraction and HPLC (High pressure liquid chromatography) [49]. Christensen et al. [50] obtained skin biopsies from 13 NSF patients who had glomerular filtration rate (GFR) <30 mL/min who had all received i.v. GBCAs. Total Gd concentrations in biopsies from affected skin areas, determined by ICP-MS, showed a mean value of 71.4 µg/g dry weight, which was significantly increased as compared with 10.2 µg/g dry weight in unaffected areas. Serum Gd levels in these NSF patients were 4.8 ng/mL. In skin biopsies from controls not treated with GBCAs, Gd concentrations were <0.1 µg/g dry weight and serum Gd < 0.2 ng/mL.

## 6. Prevention and Treatment of Gd-Related Side Effects

In order to reduce the occurrence of side effects of GBCAs it is important to be aware of the risk factors, screen individuals at risk and avoid administration of Gd-agents if alternative procedures for patients at risk can be performed. Although the importance of repeated doses of linear GBCAs for development of side effects are well established, some radiologists consider it uncertain whether any important tissue accumulation can be caused by repeated doses of the macrocyclic agents. Further studies are therefore required to solve this question. Routines with registration of cumulative doses, although recommended, are not yet generally adopted, despite the fact that in many cases repeated GBCA-assisted MRI procedures are performed. When detailed records of doses and compounds are used, it will allow calculation of cumulative doses. It would be of value to perform additional investigations on the relationship between cumulative doses of the various GBCAs and occurrence of side effects and if correlations are found, they may form a foundation for future recommendations of special precautions when repeated GBCA administrations are performed.

Several chelating agents can increase the excretion of GBCAs if administered simultaneously or immediately after the injection of GBCA [49,50]. However, there is a decrease with time in the efficacy of such chelation and it is much more difficult to mobilize gadolinium deposits that occur several days after the GBCA injection. Early administration of chelates may, therefore, be considered a possible preventive strategy in selected patients undergoing GBCA procedures. However, more basic information is needed before such recommendations can be issued.

Based on animal experiments a hydroxypyridone derivative [51] and the iron chelator deferoxamine appear promising [52,53], but clinical studies are sparse or lacking. Based on a case report, Leung et al. [54] concluded that the efficacy of deferoxamine therapy (1000 mg/d), although it increased urinary Gd clearance significantly, was too ineffective to remove sufficient amounts of Gd from blood and organs. They observed practically unchanged serum levels of about 1.5 µg/L. However, since the Gd-deferoxamine chelate is presumed to be dialyzable, combination of dialysis and chelation seems to be a promising option in cases with severe side effects. Based on experimental studies, combination of a hydroxypyridone derivative and hemoperfusion has been proposed as an efficient approach [55], but human studies are lacking. Like iron (Fe^3+^), gadolinium (Gd^3+^) can be considered a hard-to-bordeline metal with highest affinity to multidentate borderline electron donors containing nitrogen and oxygen groups [56], justifying further studies on the hydroxypyridone derivatives as Gd-antidotes.

## 7. Conclusions

The specific properties of Gd make it suitable for certain applications in medicine, being the base for chelates administered to patients as a contrast agent in magnetic resonance imaging (MRI). Such Gd-chelates have been used for more than thirty years. During the past decades, increasing knowledge about the potential harmful effects of Gd-chelates in patients with severe renal dysfunction has been gathered. In such patients there is a risk for a potentially lethal disease, nephrogenic systemic fibrosis. Restricting the use of Gd-chelates in persons with severely impaired renal function have decreased the occurrence of this toxic effect in the last decade. There has also been an increasing awareness of the fact that Gd is retained in the body, even in patients without renal dysfunction. This retention/deposition is apparently related to the cumulative number of doses given and also to the chemical structure of the chelate. As for therapeutic removal of harmful Gd deposits, traditional chelators seem inefficient, whereas a hydroxypyridone derivative combined with hemoperfusion appears promising.
